# Monensin Improves the Effectiveness of *meso*-Dimercaptosuccinate when Used to Treat Lead Intoxication in Rats

**DOI:** 10.1289/ehp.8279

**Published:** 2005-09-29

**Authors:** Shawn A. Hamidinia, Warren L. Erdahl, Clifford J. Chapman, Gregory E. Steinbaugh, Richard W. Taylor, Douglas R. Pfeiffer

**Affiliations:** 1 Departments of Molecular and Cellular Biochemistry, Ohio State University, Columbus, Ohio, USA; 2 Department of Chemistry and Biochemistry, University of Oklahoma, Norman, Oklahoma, USA

**Keywords:** chelation therapy, DMSA, heavy metal intoxication, ICP mass spectroscopy, ionophores, monensin, Pb, Pb^2+^ transport, trace metal cations

## Abstract

Among divalent cations, the ionophore monensin shows high activity and selectivity for the transport of lead ions (Pb^2+^) across phospholipid membranes. When coadministered to rats that were receiving *meso*-dimercaptosuccinate for treatment of Pb intoxication, monensin significantly increased the amount of Pb removed from femur, brain, and heart. It showed a tendency to increase Pb removal from liver and kidney but had no effect of this type in skeletal muscle. Tissue levels of several physiologic (calcium, cobalt, copper, iron, magnesium, manganese, molybdenum, zinc) and nonphysiologic (arsenic, cadmium, chromium, nickel, strontium) elements were also determined after the application of these compounds. Among the physiologic elements, a number of significant changes were seen, including both rising and falling values. The size of these changes was typically around 20% compared with control values, with the largest examples seen in femur. These changes often tended to reverse those of similar size that had occurred during Pb administration. Among the nonphysiologic elements, which were present in trace amounts, the changes were smaller in number but larger in size. None of these changes appears likely to be significant in terms of toxicity, and there were no signs of overt toxicity under any of the conditions employed. Monensin may act by cotransporting Pb^2+^ and OH^–^ ions out of cells, in exchange for external sodium ions. The net effect would be to shuttle intracellular Pb^2+^ to extracellular dimercaptosuccinic acid thereby enhancing its effectiveness. Thus, monensin may be useful for the treatment of Pb intoxication when applied in combination with hydrophilic Pb^2+^ chelators.

Several polyether ionophores, including A23187, ionomycin, monensin, and nigericin, have been shown to transport lead ions (Pb^2+^) across phospholipid bilayers and to form stable complexes with Pb^2+^ in homogeneous solution (Erdahl et al. 2000; Hamidinia et al. 2002, 2004). Among this group, the order of transport activity is ionomycin > nigericin > monensin > A23187, whereas the order of selectivity, compared with other divalent cations, is nigericin > monensin > ionomycin > A23187. Ionomycin can be used to load cultured cells and to deplete them of Pb^2+^ (Erdahl et al. 2000), and effects of monensin on Pb dynamics in rats have also been investigated (Hamidinia et al. 2002). In the latter area it was found that simultaneous administration of monensin in feed and Pb^2+^ in drinking water lowers the prevailing concentration of Pb in blood and decreases the accumulation of Pb in several soft tissues and in bone. Feeding monensin after a period of Pb^2+^ administration was furthermore effective at accelerating the clearance of Pb from brain, liver, kidney and bone, with the monensin-related increment being found in feces, as opposed to the Pb excreted spontaneously, which included a component found in urine (Hamidinia et al. 2002).

The actions of monensin on Pb dynamics in rats suggest the possibility of using ionophores in the treatment of human Pb intoxication. At present, the ongoing and widespread problem of Pb intoxication is treated first by removing the individual from the Pb-contaminated environment and thereafter by administering a Pb^2+^ chelating agent. *meso*-Dimercaptosuccinic acid (DMSA) is the most commonly used agent at present, in part because it can be administered orally and is well tolerated (Cory-Slechta 1988; Graziano et al. 1985). DMSA is a water-soluble compound that forms a strong complex with Pb^2+^ in blood, which is thereafter secreted via the kidney. EDTA and other chelating agents are used in a similar way to treat Pb intoxication (Goyer et al. 1995; Llobet et al. 1990). In all cases, several cycles of chelator administration given over a period of months are required to produce an adequate and durable reduction of Pb in blood. This is because the blood pool is equilibrated with Pb in other internal compartments, such that a blood pool is reestablished as Pb is mobilized from the other compartments after a cycle of chelator administration (Graziano 1986, 1993; Graziano et al. 1985, 1988). The repetitive treatments and extended time frames are problematic, particularly when treating children, because important aspects of Pb toxicity arise to a greater degree if Pb is present before development is completed. The propensity of Pb to lower IQ (intelligence quotient) and otherwise interfere with functions of the central nervous system is perhaps the most important of these aspects (Goyer 1993). An uneven effectiveness of water-soluble chelators at removing Pb from particular organs is another problem with existing protocols for the treatment of Pb intoxication, with bone (Castellino and Aloj 1964; Cory-Slechta 1988; Gerhardsson et al. 1999; Smith et al. 2000) and brain (Cremin et al. 1999; Seaton et al. 1999; Smith et al. 1998) being particularly difficult to free of the accumulated cation. In addition, chelators can provoke an undesirable redistribution of Pb among soft tissues and between bone and soft tissues (Cory-Slechta et al. 1987).

Within this context of less than ideal treatments for Pb intoxication and the recent discovery that several carboxylic acid ionophores transport Pb^2+^ with high specificity, we are seeking to determine if some of these compounds might be used together with the traditional chelators to improve the effectiveness of existing treatment protocols. The present report describes the effectiveness of coadministering monensin and DMSA in this regard, using rats as an experimental model.

## Materials and Methods

### Treatment of experimental animals.

We used male Sprague-Dawley rats throughout the study. They were treated humanely and with regard to the alleviation of suffering. The rats were housed at the College of Medicine, Ohio State University, in animal facilities approved by the Association for Accreditation of Laboratory Animal Care. A 12-hr light/dark cycle, dual housing in plastic cages, and conditions of constant temperature and humidity were employed. We allowed 1 week for acclimation before the experimental protocol began. During this period rats were fed a standard laboratory chow, whereas the AIN-93M diet containing 0.5% calcium (normal chow; Harlan Teklad, Madison, WI) was employed thereafter. During the administration of Pb^2+^ and/or monensin and DMSA, water and feed were provided *ad libitum*, and records of consumption were maintained together with periodic measurements of body weight.

Initially the rats weighed 245–255 g. They were divided into five groups of eight, and the administration of Pb^2+^ was begun. It was provided at 100 ppm in the drinking water (0.48 mM) and was in the form of Pb(acetate)_2_. The water was rendered slightly acidic with acetic acid to prevent the precipitation of PbCO_3_ (lead carbonate) (Bogden et al. 1995). As a point of reference, after several days this regimen produces a circulating Pb level of 1.1 μM in rats (22 μg/dL) (Hamidinia et al. 2002), which is higher by about a factor of 2 than the value of 0.5 μM (10 μg/dL), which is often taken to be the toxic threshold for Pb in children.

After 3 weeks, one group of rats was sacrificed to determine the Pb content of organs at that time (the Pb-loaded group), whereas the remaining groups thereafter received water that did not contain Pb^2+^. One of these groups received the normal chow (no-treatment group), another received this chow containing 100 ppm monensin (monensin group), a third group received normal chow and DMSA administered by oral gavage (DMSA group), and a final group received the chow containing monensin and DMSA delivered by oral gavage (monensin plus DMSA group). When administered, the 50 mg/kg dose of DMSA was given every other day. The solution volume administered varied between approximately 0.25 and 0.45 mL as body weight increased so as to maintain the prescribed dose. The DMSA solution was freshly prepared by dissolving the compound in 5% NaHCO_3_ (sodium bicarbonate). All treatments were continued for 3 weeks beyond the time when the administration of Pb^2+^ had been discontinued. The rats were then sacrificed by the injection of excess Nembutal (Abbott Laboratories, North Chicago, IL) and were subsequently perfused briefly with HEPES-buffered 0.9% NaCl, via the left ventricle, to remove blood from the organs. After this, organs and tissues of interest were removed and stored at –20°C.

### Determination of Pb and other elements by inductively coupled plasma mass spectroscopy.

All aspects of the analytical procedures were conducted in laminar flow hoods within the Microscopic and Chemical Analysis Research Center at Ohio State University. The frozen organs were first thawed and weighed and were then digested in 5.00 mL of trace-metal–grade concentrated nitric acid. One intact kidney, the entire brain, the entire heart, and the left femur from each animal were analyzed. For liver, a portion of the right lateral lobe weighing approximately 2 g was analyzed, whereas for muscle it was a similar-sized portion of the left biceps femoris. Digestion was conducted in acid-cleaned quartz vials that were contained in a Teflon liner that was itself contained in a closed high-pressure vessel (Milestone Inc). The Teflon liners contained 10 mL of a 6% H_2_O_2_ solution, in which the quartz vial was partially immersed, so as to minimize the rise in internal pressure that occurs as digestion proceeds. Samples were heated to 180°C in a microwave apparatus (Ethos Plus; Milestone Inc., Shelton, CT). Temperature programming provided for a linear rise to that value over a 10-min period, a holding period of 10 min, and a 15-min cool-down period. Sample blanks containing no tissue were generated in the same way, as were occasional tissue samples that were spiked with gallium (used to estimate overall recovery from the procedure). After digestion, the samples were transferred quantitatively to Nalgene LDPE Boston round bottles (Fisher Scientific, Pittsburgh, PA), diluted to 50.0 mL with NANOpure water (Barnstead/Thermolyne, Dubuque, IA), and then stored at room temperature.

Subsequent steps were carried out within 1 month. A cocktail of three internal standards (bismuth, scandium, and rhodium) made from CPI International (Santa Rosa, CA) peak performance standards was added to each sample (100 μL in 10.0 mL) so as to give a 10 ppb concentration of each standard. The signals arising from these standards were used to correct data for variations in instrument performance that occur while a set of samples is being analyzed. A set of calibration standards, similar in composition to the unknown samples, was prepared for each of the six tissues and used to convert data output from the instrument into units of concentration. These were prepared using SPEX Certi Prep (Metuchen, NJ) multielement standards with appropriate adjustments made using single-element standards obtained from CPI International. The samples were analyzed using a Thermo Finnigan magnetic sector inductively coupled plasma (ICP) mass spectrometer (Thermo Electron Corporation, Waltham, MA), which is capable of resolutions > 0.005 atomic mass units. The data were expressed first in units of parts per billion and were subsequently converted to units of nanomoles per gram wet weight of tissue. Replicate analysis of single samples showed that deviation arising from analysis per se was on the order of ± 2%, whereas overall recovery of unknowns was approximately 98–101%. Values obtained from sample blanks and standard solution samples that had been carried through the entire procedure showed that potential errors arising from contamination in reagents, leaching/absorption of materials from labware, and so on, could be ignored for most elements. Nevertheless, duplicate blanks were run with each set of samples, and the values obtained were subtracted from the standard and experimental values during data analysis.

Pb levels in the various tissues were expressed as means ± SE and thereafter by the percentage change in mean values. Comparisons between groups were made using Student’s *t*-test with differences reported as significant for *p*-values < 0.05. For elements other than Pb, we asked if mean values differed between treatment groups, and the two-tailed test was employed accordingly. In the case of Pb, previous work indicated that all treatments would enhance depletion and would not be expected to elevate the levels observed. According, when analyzing the Pb data, we asked if treatment in question lowered Pb, relative to the appropriate control, and the one-tailed test was employed (Christenson and Stoop 1986).

## Results

### Levels of Pb and other elements after Pb administration.

Monensin is highly selective for the transport of Pb^2+^, compared with other divalent cations (Hamidinia et al. 2002), and this is one of its characteristics that led us to test this ionophore for possible use in conjunction with DMSA for the treatment of Pb intoxication. That is to say, it seemed possible that monensin might aid in the delivery of intracellular Pb^2+^ to the circulating chelator without greatly perturbing the intracellular level of other cations. On the other hand, the available selectivity data were obtained using a model transport system based on phospholipid vesicles and did not include all cations of possible interest. Thus, it was not clear initially to what extent the selectivity for Pb^2+^ would manifest *in vivo*, or if cations having a physiologic role, but not yet considered in terms of selectivity, might also be perturbed. Accordingly, we examined the tissue levels of several physiologic elements (Ca, cobalt, copper, iron, manganese, magnesium, molybdenum, zinc) using ICP mass spectrometry as the detection modality. We also examined levels of several elements having no physiologic role (arsenic, cadmium, chromium, nickel, strontium) to determine if the ionophores actions are specific for Pb among a known group of potential toxins.

In Table 1 we compare the endogenous levels of these cations as reported by Naveh et al. (1987) with those we found at the end of the 3-week Pb loading period. As expected, Pb rose markedly in all organs examined, but there were large differences in the absolute levels attained. Specifically, the order of Pb levels observed was femur > kidney > liver > brain > heart > skeletal muscle, with the highest value exceeding the lowest one by approximately 2.5 × 10^3^. These findings are similar to those reported by others (Bogden et al. 1992; Han et al. 1996; Naveh et al. 1987).

Among the other elements considered, comparisons were possible for Ca, Cu, Fe, Mg, Mn, and Zn, but not for Co, Mo, As, Cd, Cr, Ni, and Sr, because the reference study did not include data for the latter group. Among the former group, three large variations were seen: the level of Ca in kidney and the levels of Cu and Mn in femur. In all three cases, the values reported here are about 10% of the reference values, and the differences are statistically significant. Although there are several possible reasons for variations of this magnitude, as further considered in the “Discussion,” we doubt that they reflect authentic effects of Pb administration and have used our present values when interpreting other aspects of the data. Among the other organs and elements considered, variations were much smaller. Reference values for Fe in kidney and Mn in skeletal muscle are higher than what we found after Pb loading, by about a factor of 2, and these differences were also statistically significant. Regarding the other variations, many were not significantly different, whereas others were, even when the extent of variation was quite small (Table 1).

### Depletion of previously accumulated Pb.

**Figure 1:**
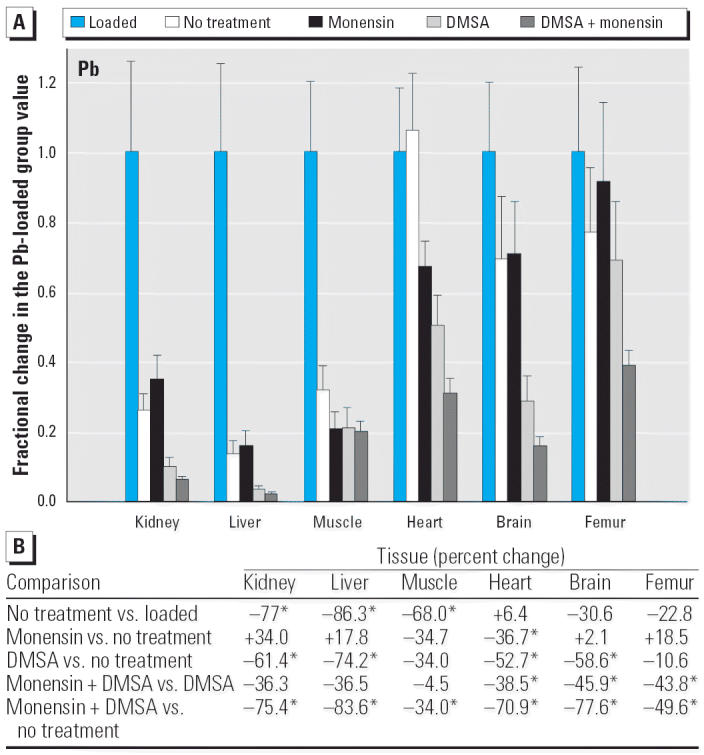
(*A*) Fractional changes (+ SE) in tissue levels of Pb produced by selected treatments. Rats were treated as described in “Materials and Methods.” Pb levels observed for the Pb-loaded group are presented in Table 1; values for the Pb-loaded group were set to 1.0 to aid in normalizing values of other treatment groups. (*B*) Percent changes in mean values calculated using the same data shown in (*A*). *Statistically significant (*p* < 0.05).

To express the relative effectiveness of the four treatments that were applied after the period of Pb administration, we calculated the fraction of the Pb loaded value that remained when the treatment period was complete (3 weeks) and compared these values with each other and with the endogenous values. Results are shown graphically and numerically in Figure 1. Two subsets are apparent among the six tissues examined: kidney, liver, and skeletal muscle compared with heart, brain, and femur. For those in the first subset, the mean Pb level fell dramatically simply in response to halting its administration, and these were fully significant declines compared with the levels that existed at the end of the loading period (Figure 1). For this subset, monensin alone did not alter the values significantly, compared with withdrawal alone, whereas the further decrease produced by DMSA was significant in kidney and liver. The former result might not be expected, whereas the latter was expected, based on previous reports (Cory-Slechta 1988; Hamidinia et al. 2002; Jones et al. 1997; Pappas et al. 1995). Regarding monensin used together with DMSA, the mean values in kidney and liver were lower than those produced by DMSA alone, but the *p*-values (0.08 in kidney, 0.14 in liver) fell short of the usual threshold of significance (0.05).

For the other subset of tissues (heart, brain, and femur), halting administration alone did not significantly lower the level of Pb during the 3-week test period. Compared with the values obtained by withdrawal, the effects of monensin alone were again not significant, except in the case of heart, where the ionophore did produce a lower value. Regarding DMSA alone, this treatment lowered Pb in heart and brain but did not decrease Pb in bone, again, as might be expected (Cory-Slechta 1988; Gerhardsson et al. 1999; Jones et al. 1997; Smith et al. 2000). Of greater interest, in all three tissues, monensin plus DMSA lowered mean Pb values more noticeably than was seen in the other tissues, compared with the effect of DMSA alone, and these differences were statistically significant (Figure 1). Furthermore, the decrease produced by monensin plus DMSA in bone was significant compared with withdrawal alone, and this was also true in the five other tissues (Figure 1).

### Effects of monensin alone on the levels of other elements.

**Figure 2:**
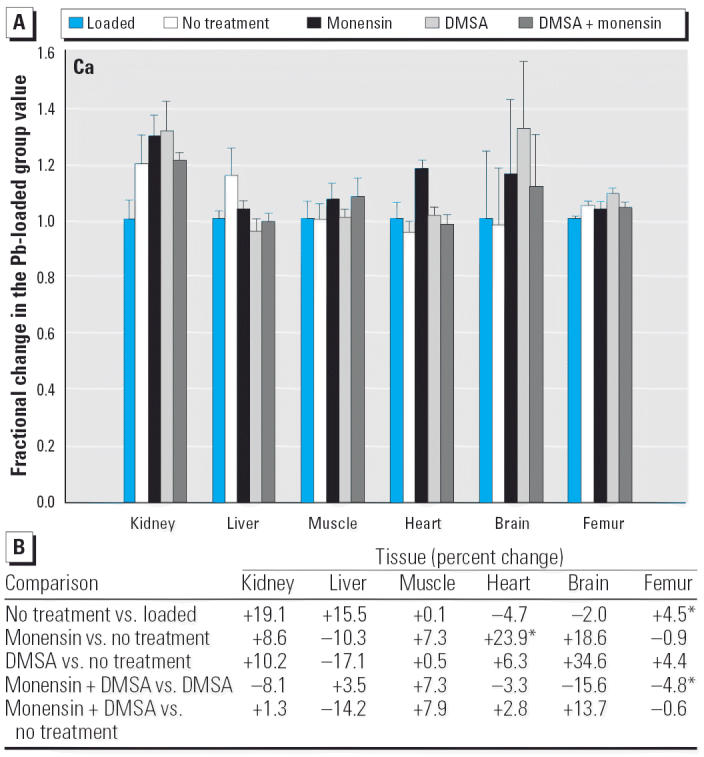
(*A*) Fractional changes (+ SE) in tissue levels of Ca occurring during treatment for Pb intoxication. For details, see “Materials and Methods” and Table 1; values for the Pb-loaded group were set to 1.0 to aid in normalizing values of other treatment groups. (*B*) Percent changes in mean values calculated using the same data shown in (*A*). *Statistically significant (*p* < 0.05).

**Figure 3:**
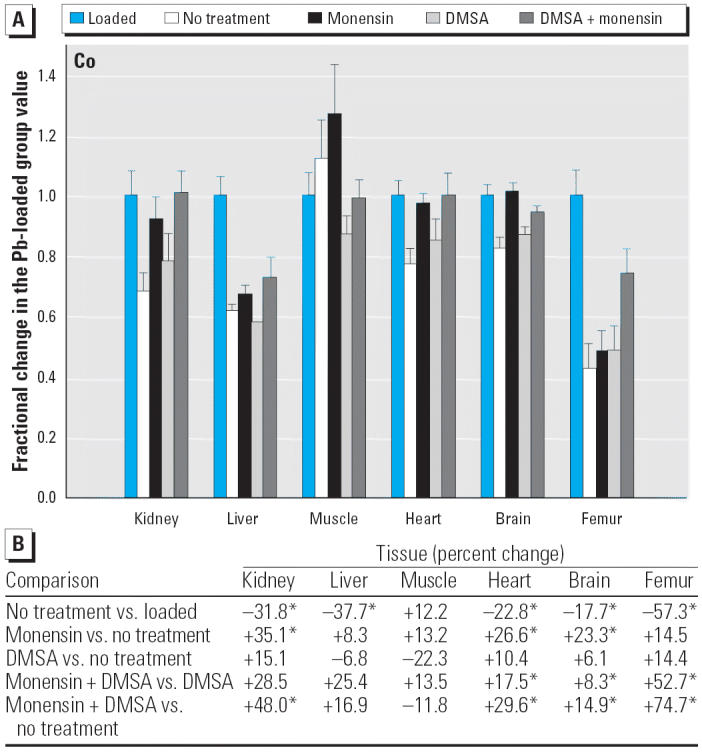
(*A*) Fractional changes (+ SE) in tissue levels of Co occurring during treatment for Pb intoxication. For details, see “Materials and Methods” and Table 1; values for the Pb-loaded group were set to 1.0 to aid in normalizing values of other treatment groups. (*B*) Percent changes in mean values calculated using the same data shown in (*A*). *Statistically significant (*p* < 0.05).

**Figure 4:**
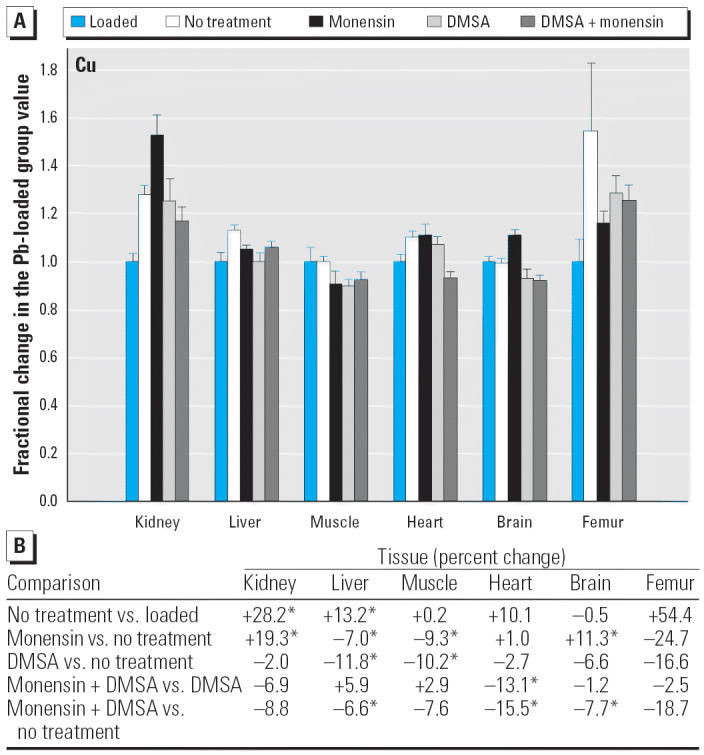
(*A*) Fractional changes (+ SE) in tissue levels of Cu occurring during treatment for Pb intoxication. For details, see “Materials and Methods” and Table 1; values for the Pb-loaded group were set to 1.0 to aid in normalizing values of other treatment groups. (*B*) Percent changes in mean values calculated using the same data shown in (*A*). *Statistically significant (*p* < 0.05).

**Figure 5:**
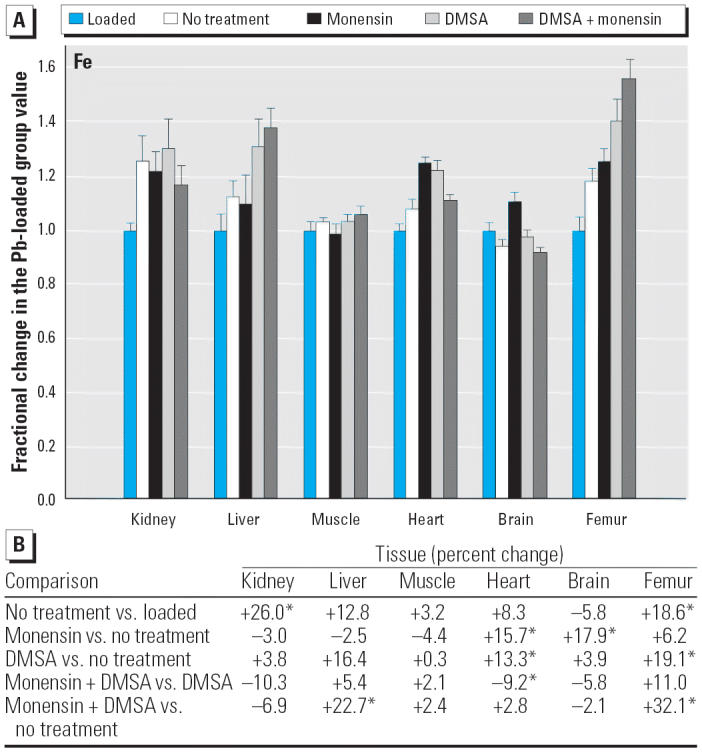
(*A*) Fractional changes (+ SE) in tissue levels of Fe occurring during treatment for Pb intoxication. For details, see “Materials and Methods” and Table 1; values for the Pb-loaded group were set to 1.0 to aid in normalizing values of other treatment groups. (*B*) Percent changes in mean values calculated using the same data shown in (*A*). *Statistically significant (*p* < 0.05).

**Figure 6:**
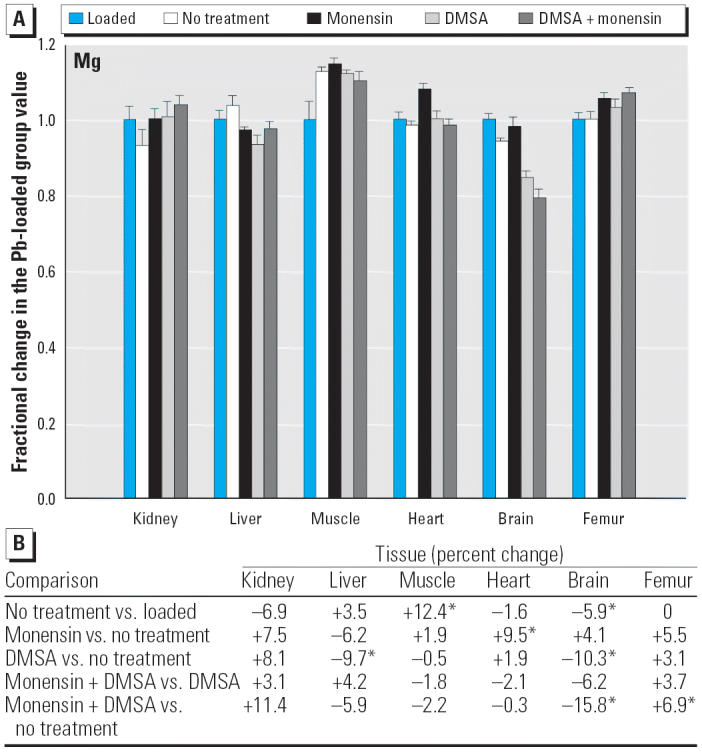
(*A*) Fractional changes (+ SE) in tissue levels of Mg occurring during treatment for Pb intoxication. For details, see “Materials and Methods” and Table 1; values for the Pb-loaded group were set to 1.0 to aid in normalizing values of other treatment groups. (*B*) Percent changes in mean values calculated using the same data shown in (*A*). *Statistically significant (*p* < 0.05).

**Figure 7:**
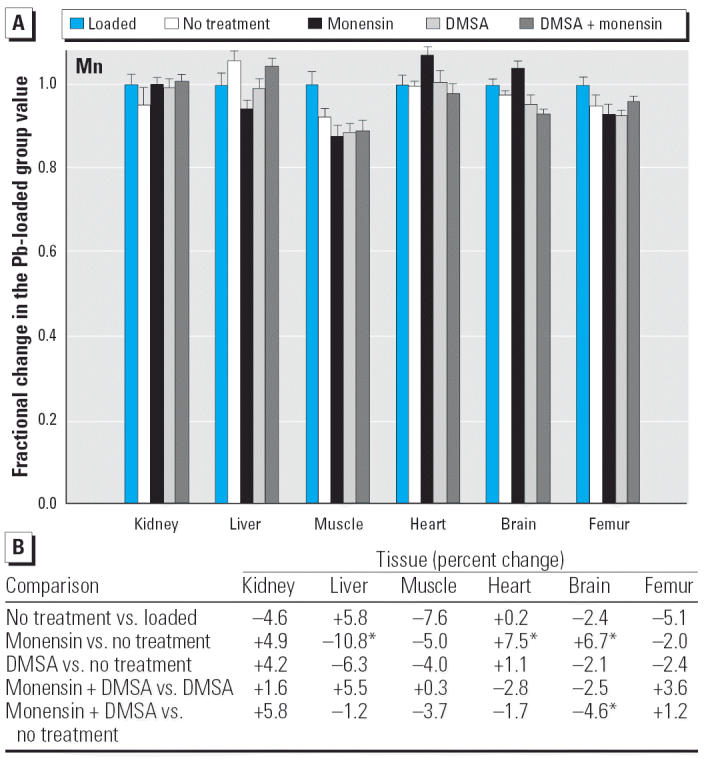
(*A*) Fractional changes (+ SE) in tissue levels of Mn occurring during treatment for Pb intoxication. For details, see “Materials and Methods” and Table 1; values for the Pb-loaded group were set to 1.0 to aid in normalizing values of other treatment groups. (*B*) Percent changes in mean values calculated using the same data shown in (*A*). *Statistically significant (*p* < 0.05).

**Figure 8:**
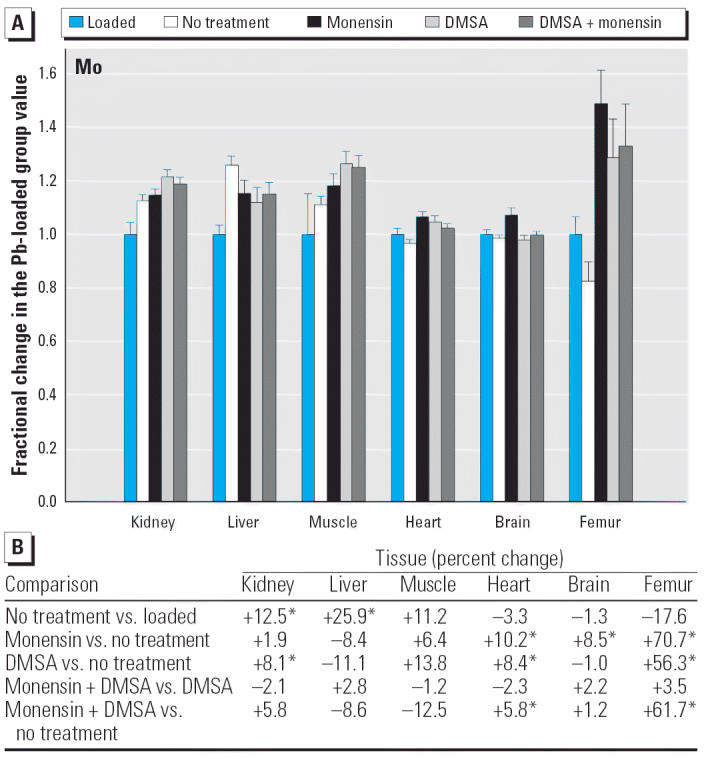
(*A*) Fractional changes (+ SE) in tissue levels of Mo occurring during treatment for Pb intoxication. For details, see “Materials and Methods” and Table 1; values for the Pb-loaded group were set to 1.0 to aid in normalizing values of other treatment groups. (*B*) Percent changes in mean values calculated using the same data shown in (*A*). *Statistically significant (*p* < 0.05).

**Figure 9:**
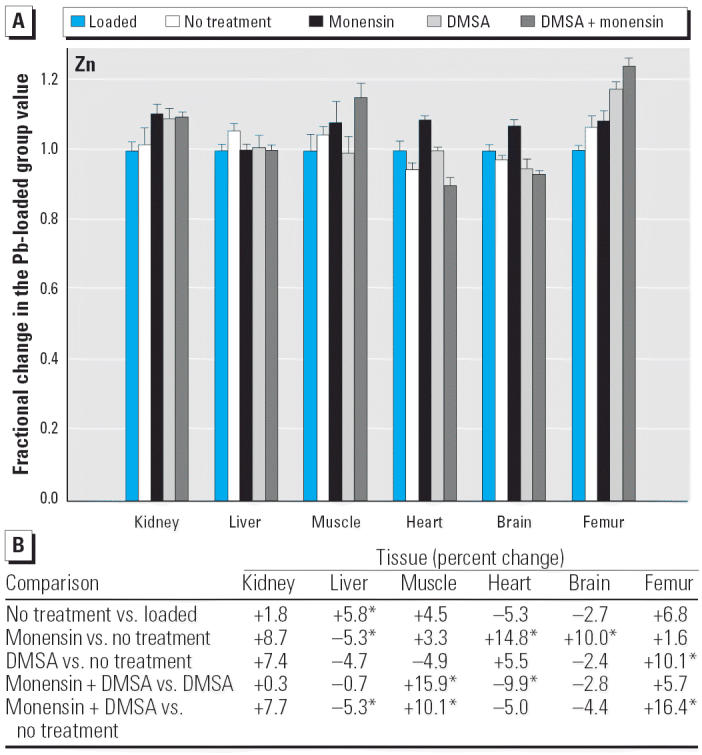
(*A*) Fractional changes (+ SE) in tissue levels of Zn occurring during treatment for Pb intoxication. For details, see “Materials and Methods” and Table 1; values for the Pb-loaded group were set to 1.0 to aid in normalizing values of other treatment groups. (*B*) Percent changes in mean values calculated using the same data shown in (*A*). *Statistically significant (*p* < 0.05).

**Figure 10:**
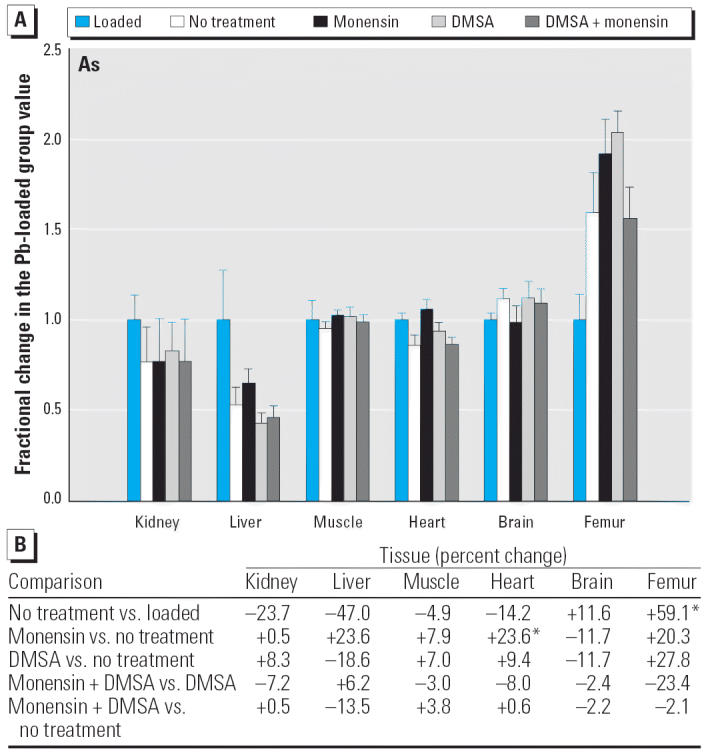
(*A*) Fractional changes (+ SE) in tissue levels of As occurring during treatment for Pb intoxication. For details, see “Materials and Methods” and Table 1; values for the Pb-loaded group were set to 1.0 to aid in normalizing values of other treatment groups. (*B*) Percent changes in mean values calculated using the same data shown in (*A*). *Statistically significant (*p* < 0.05).

**Figure 11:**
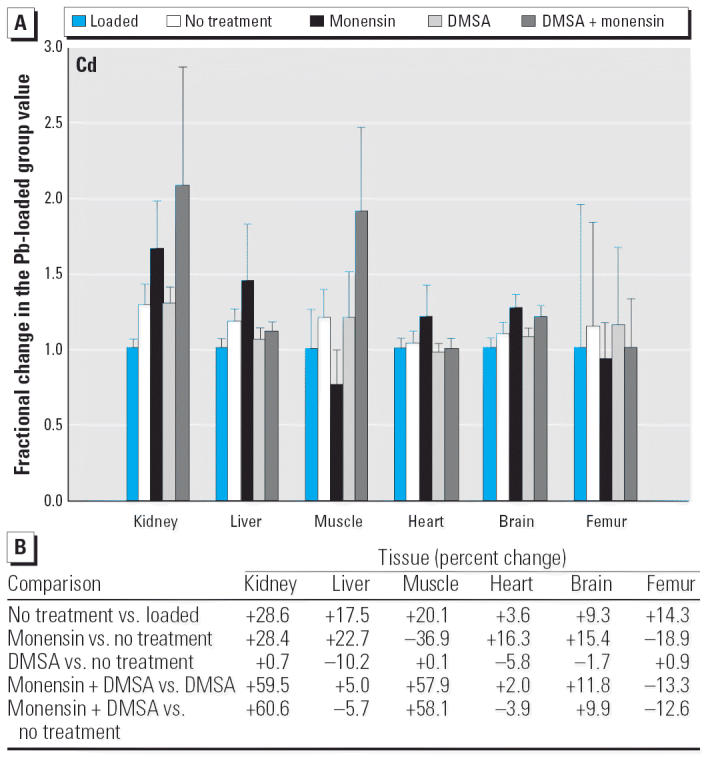
(*A*) Fractional changes (+ SE) in tissue levels of Cd occurring during treatment for Pb intoxication. For details, see “Materials and Methods” and Table 1; values for the Pb-loaded group were set to 1.0 to aid in normalizing values of other treatment groups. (*B*) Percent changes in mean values calculated using the same data shown in (*A*).

**Figure 12:**
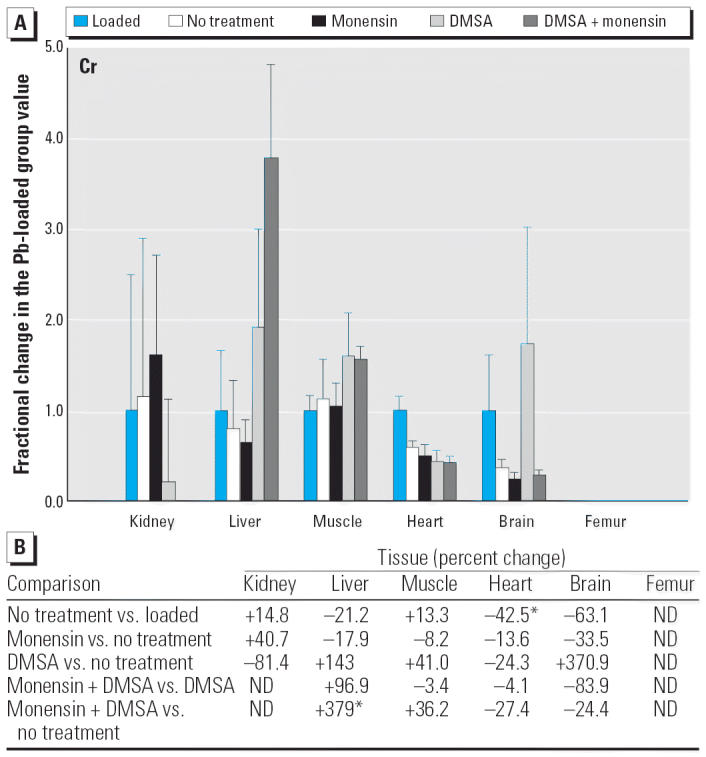
(*A*) Fractional changes (+ SE) in tissue levels of Cr occurring during treatment for Pb intoxication. For details, see “Materials and Methods” and Table 1; values for the Pb-loaded group were set to 1.0 to aid in normalizing values of other treatment groups. Where data are missing, levels of Cr were too low to be determined relative to the levels in blanks that arose as contaminants. (*B*) Percent changes in mean values calculated using the same data shown in (*A*). ND, not detected. *Statistically significant (*p* < 0.05).

**Figure 13:**
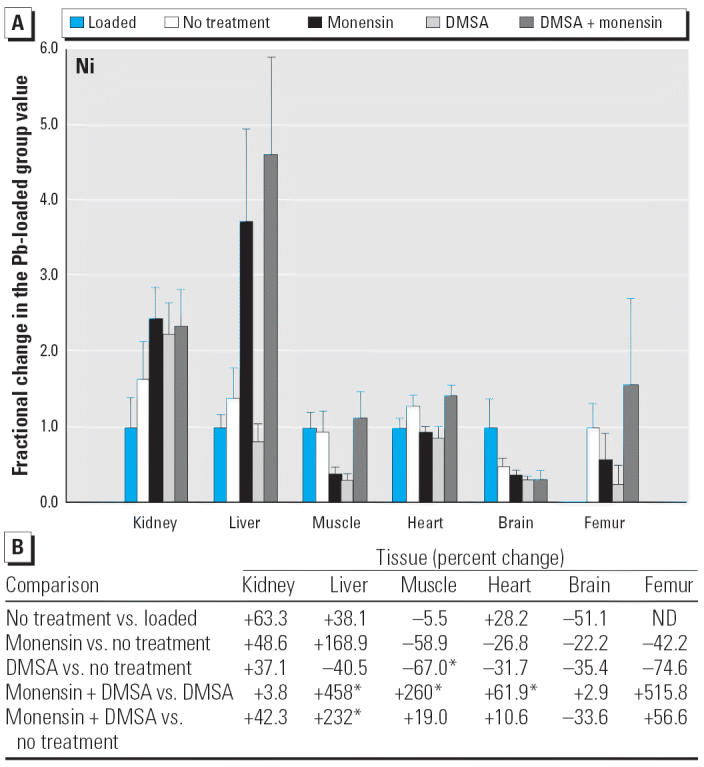
(*A*) Fractional changes (+ SE) in tissue levels of Ni occurring during treatment for Pb intoxication. For details, see “Materials and Methods” and Table 1; values for the Pb-loaded group were set to 1.0 to aid in normalizing values of other treatment groups. Where data are missing, levels of Ni were too low to be determined relative to the levels in blanks that arose as contaminants. (*B*) Percent changes in mean values calculated using the same data shown in (*A*). ND, not detected. *Statistically significant (*p* < 0.05).

**Figure 14:**
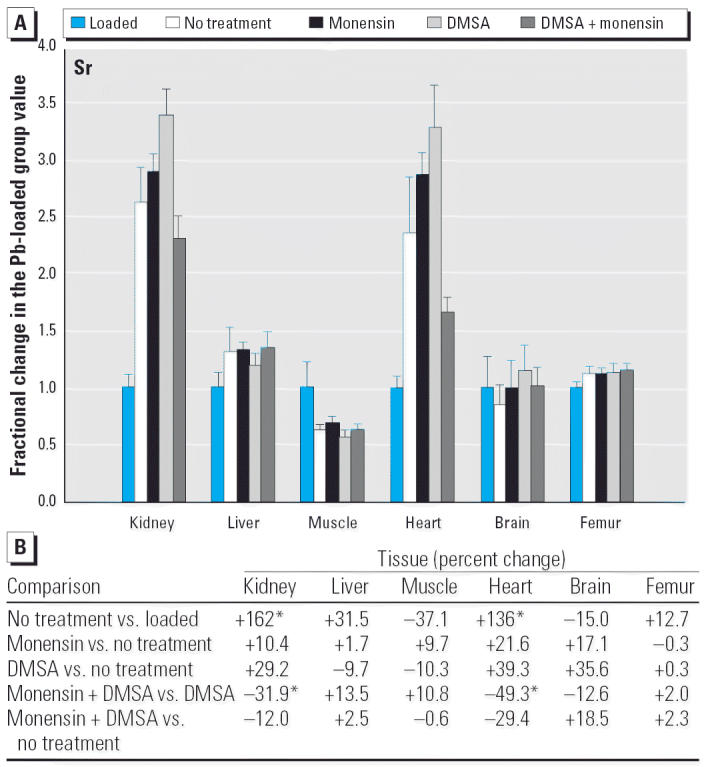
(*A*) Fractional changes (+ SE) in tissue levels of Sr occurring during treatment for Pb intoxication. For details, see “Materials and Methods” and Table 1; values for the Pb-loaded group were set to 1.0 to aid in normalizing values of other treatment groups. (*B*) Percent changes in mean values calculated using the same data shown in (*A*). *Statistically significant (*p* < 0.05).

Regarding the other elements, data are presented in Figures 2–14, which are in the same format used for Figure 1. Given that we are primarily interested in determining if monensin might be useful in the treatment of Pb intoxication, we focus first on whether or not the ionophore given alone perturbs physiologic elements. This point is of interest because such perturbations might lead to secondary forms of toxicity. Regarding Ca, monensin alone increased the level in heart by approximately 24%, compared with rats that received no treatment for accumulated Pb, but had no significant effect in the other tissues (Figure 2). By the same comparison, Co was decreased in kidney, heart, and brain, but in the no-treatment group Co fell during the 3-week period after Pb administration had ended (Figure 3). Thus, the 25–30% increases in Co produced by monensin were tending to reverse that initial decline and might therefore be viewed as advantageous. Cu was increased in kidney and brain, decreased slightly in liver and skeletal muscle, and was not changed significantly in heart and femur (Figure 4). Fe rose modestly in heart and brain but was not perturbed significantly elsewhere (Figure 5). Mg was altered only in heart (Figure 6), and Mn in liver, heart, and brain (Figure 7); these were all modest variations of around 15% or less. Changes in Mo and Zn were similarly small (Figures 8 and 9), except for the Mo level in femur, which decreased by 70% (Figure 8).

Among the nonphysiologic elements (Figures 10–14), the only statistically significant effect produced by monensin alone was in heart, where the trace level of As rose by approximately 24% (Figure 10).

### Perturbations produced by monensin in DMSA-treated rats.

Because Figure 1 indicates that monensin plus DMSA is more effective at depleting Pb than either agent used alone, it is also of interest to determine if the two agents used together perturb other elements more so than does DMSA alone and to examine perturbations produced by the combination of compounds compared with no treatment. Considering the former comparison first, and for the physiologic elements, monensin altered the levels of Ca, Co, Cu, Fe, and Zn in one or more tissues, more so than did DMSA alone, whereas the levels Mg, Mn, and Mo were not affected statistically (Figures 2–9). Among the changes of this type, all were small (on the order of < 15%), except for the level of Co in femur, which increased by 53% (Figure 3). Furthermore, the directions of these changes were such that they tended to reverse changes that arose from DMSA alone and thus might be viewed as beneficial. This is with the exception of Zn in skeletal muscle and heart (Figure 9), which was perturbed to a greater extent by monensin plus DMSA than it was by DMSA alone, although the effects were small (+16% and –10%, respectively).

Considering the nonphysiologic elements, monensin plus DMSA increased Ni substantially in liver, skeletal muscle, and heart, compared with DMSA alone (Figure 13), and likewise decreased Sr in kidney and heart (Figure 14). The changes in Sr were in the direction that corrected a perturbation produced by DMSA alone, but this was not true with Ni.

### Perturbations produced by monensin plus DMSA compared with no treatment.

Within this last area of interest, and among the physiologic elements, monensin plus DMSA produced a change in one or more tissues in all cases except Ca. Co rose in kidney, heart, and femur, but these changes were again tending to reverse declines that otherwise occurred in rats that were not treated (Figure 3). Cu fell modestly in liver, heart, and brain; Fe rose in liver and femur, whereas Zn was altered in liver, skeletal muscle, and femur (Figures 7–9). All of these changes were again small, with the exception of Mo in femur, where the increase was 62%.

Considering the nonphysiologic elements (Figures 10–14), significant changes were confined to Cr and Ni, which were both increased in liver (Figures 12 and 13).

### Effects of treatment on other parameters.

**Figure 15:**
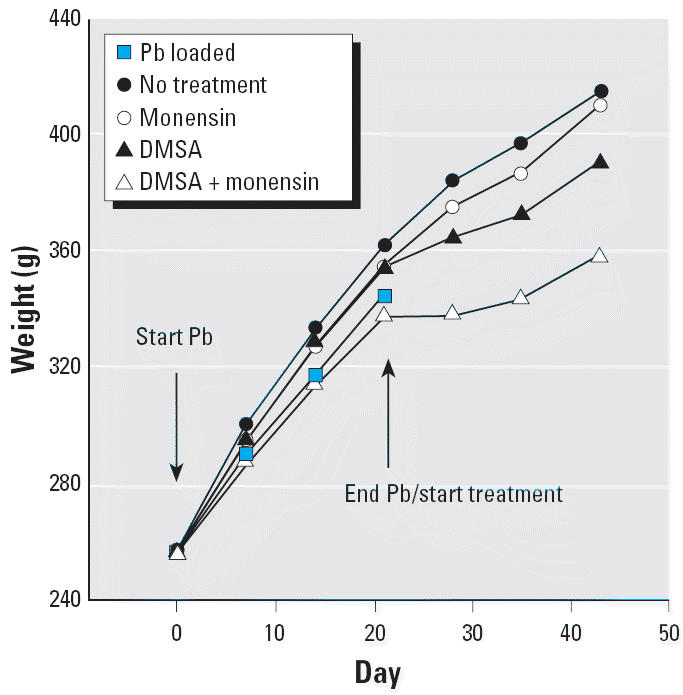
Effect of treatment on weight gain in Pb-intoxicated rats. Data are from the same rats used to obtain the data in Figures 1–14. For details, see “Materials and Methods.”

Regarding macroscopic effects of the treatment strategies investigated, there was no indication that the rats were differently stressed, as indicated by their behavior, activity level, general appearance, and the macroscopic appearance of internal structures seen during dissection. Records of weight gain throughout the experimental period are shown in Figure 15. During the period of Pb administration, when all rats were maintained in the same way, the per animal average weight of the five groups diverged such that there was a 25 g range in this parameter at the time that Pb was withdrawn. Comparing the no-treatment and the monensin-alone groups showed no significant difference in the subsequent rate of weight gain (Figure 15). For the rats receiving DMSA or DMSA plus monensin, weight gain lagged initially but then returned to a rate that was similar to that seen in the no-treatment or the monensin-alone groups. An unpleasant odor of the dimercaptide and gavage-related irritation of the gastrointestinal tract may have contributed to these lags in weight gain by discouraging eating.

## Discussion

The results of the present study demonstrate that monensin plus DMSA is more effective than DMSA alone in terms of depleting rats of previously accumulated Pb, and that the combination of agents is effective in all organs/tissues examined. Comparing the agents used in combination with DMSA alone, monensin significantly improved the outcome in heart, brain, and femur and showed a tendency to do this in kidney and liver. The improved clearance seen in heart, brain, and femur is notable because withdrawal of Pb alone is not very effective at reducing Pb in these tissues, because DMSA used alone is less effective in these tissues than in some others, and because the actions of Pb in heart, brain, and bone account for significant aspects of Pb patho-physiology. Thus, in heart, Pb accumulation generates a set of effects that are analogous to those produced by various forms of human cardiac disease (Williams et al. 1983). In brain, Pb effects manifest at low overall levels and include cognitive impairment and reduced IQ (Goyer 1993; Goyer et al. 1995; Rice 1996). Bone Pb is problematic because this pool is large and is the main source of Pb that reestablishes an elevated blood concentration after treatment by existing methods (Cory-Slechta 1988; Leggett 1993; O’Flaherty 1991, 1995; O’Flaherty et al. 1998). Bone Pb can furthermore be mobilized together with Ca during aging and pregnancy to produce toxic effects even when there is no ongoing exposure to Pb from the environment. Thus, it seems worthwhile to further explore the possibility of using ionophores together with the traditional agents in the clinical treatment of Pb intoxication.

Potential toxicity of the ionophore itself must be considered when contemplating a clinical application. There are no data available on the toxic actions of monensin in humans; however, the compound has long been administered to a variety of animal species that are used to produce food in agriculture. This practice arose because monensin is an anticoccidial agent, because it promotes growth, and because it is easily administered in feed as was done during this study (Ruff 1982; Shumard and Callender 1968; Walker et al. 1980). The level of 100 ppm used here is typical of that used in agriculture and is well below the level of 200 ppm where toxic actions are first seen in rats (Ruff 1982; Todd et al. 1984). To these points we can now add that monensin plus DMSA also produces no overt toxicity in rats and has little effect on growth beyond that seen with DMSA alone (Figure 15).

To further examine the potential for toxicity, we determined how tissue levels of other elements changed during a period of Pb administration and during our attempts to remove it. Among the elements having a physiologic role, no situation was found in which the level of an element was markedly decreased compared to that which existed when Pb administration had been completed and treatment was about to begin. The closest we saw to this type of situation was with Co, where levels tended to decline after removal of Pb from the drinking water. The various treatments used to remove Pb either had little further effect or tended to restore Co to its pretreatment value (Figure 3).

Likewise, we did not encounter examples in which the levels of a physiologic element rose markedly, compared with a pretreatment value, as a result of any approach taken to remove Pb. Cu, Fe, Mo, and Zn rose variously in some tissues by as much as 50%, and there were a number of smaller changes (increases and decreases) where statistical significance could be demonstrated (Figures 2–9). It is difficult to say if any of these are of consequence because data describing the range of values found in normal rats as a function of strain, diet, age, and so on, are sparse. It also is not clear to what extent a “normal value” may be decreased or increased before giving rise to symptoms of deficiency or overload toxicity, respectively.

Some further insight into these types of questions is obtained by examining Table 1, which shows tissue levels of physiologic elements in rats that had received Pb and levels in rats that had not been exposed. We selected data from unexposed rats reported by Naveh et al. (1987) for comparison because the rats they used were of a similar age and dietary history. As seen globally in Table 1, the size of variations that were identified are similar to those that arose during attempts to remove Pb after it had accumulated. This supports our view that the changes in physiologic element levels that were seen during treatment for Pb intoxication are of little consequence. This is with the exception of Ca levels in kidney and the levels of Cu and Mn in femur. There, the same argument cannot be employed because the normal values reported by Bogden and colleagues (Naveh et al. 1987) fare about 10-fold higher than what we found after Pb administration. These large variations may reflect methodologic problems arising during calibration or data analysis. We rechecked our own methods upon seeing these differences and note that our values are close to those reported by others (Oishi et al. 2000; Seaborn and Nielsen 2002a, 2002b).

Another type of potential toxicity to consider in contemplating the use of monensin to treat Pb intoxication is the possibility that other toxic elements present in the individual might be perturbed in such way as to enhance their toxicity, even though the toxicity from Pb has been abated. Among the five toxic elements examined, we found scattered examples where one or more of the treatment strategies increased the level of a toxin in one or more of the tissues (Figures 10–14). When observed, these perturbations were sometimes much larger than what was seen with physiologic elements (e.g., the 2- to 4-fold rise of Cr and Ni in liver during treatment with monensin plus DMSA), supporting the prospects for increased toxicity. On the other hand, none of the non-physiologic elements was being administered beyond the trace quantities presumably present in diet, water, and the general environment provided to the rats. Accordingly, the levels of these were very low (Table 1) even when they had increased during attempts to removed Pb. Thus, it is again difficult to judge if these changes are meaningful in terms of toxicity without conducting additional studies in which the element of interest is purposely administered. The same can be said for the scattered examples where certain treatments for Pb intoxication decreased the level of another toxin (e.g., Cr and Ni in brain and As in liver).

Interest is growing in the combined use of multiple chelating agents for the treatment of metal intoxication (Andersen and Aaseth 2002; Kalia and Flora 2005; Link et al. 2001; Wu et al. 2003), although the success of this approach with Pb intoxication has been limited (Jones et al. 1997; Kachru et al. 2005; Kostial et al. 1999). The question then arises: How does monensin improve the effectiveness of DMSA at removing Pb from various tissues to the extent that is seen here? The multiple chelator approach seems to work best when one of the agents is fully water soluble and the other has some hydrophobic character (Andersen and Aaseth 2002; Giardina and Grady 2001). This has been explained by the so-called relay hypothesis, or shuttle hypothesis, which maintains that the more hydrophobic agent is able to bind toxic cations that are located in compartments not accessible to the hydrophilic compound and can thereafter facilitate their movement and transfer to the hydrophilic compound. Once this has occurred, the hydrophilic compound is excreted via the kidney, together with the chelated cation. Monensin is highly hydrophobic and is in fact a highly selective and efficient ionophore for Pb^2+^ (Hamidinia et al. 2002). It may then be particularly effective at shuttling Pb^2+^ to DMSA located in blood or in the interstitial fluid compartment.

**Figure 16:**
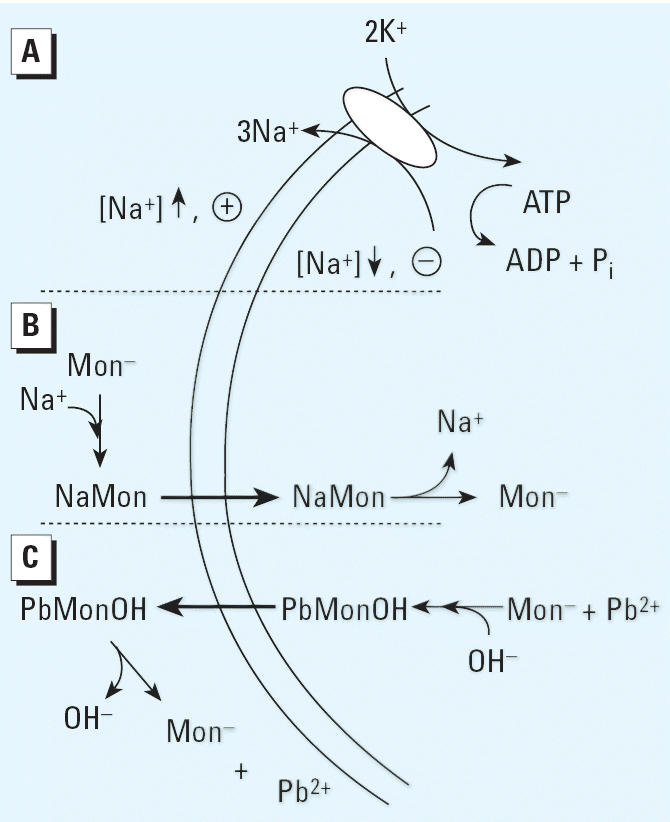
Potential relationships between the transport of Na^+^ and Pb^2+^. The two parallel curved lines represent a section of plasma membrane bounding a cell that contains Pb^2+^. To the left and right of these lines are the extra- and intracellular compartments, respectively. P_i_ indicates inorganic phosphate; “+” and “–” indicate the membrane potential orientation inside and outside the vesicle. (*A*) The formation of an electrochemical Na^+^ gradient across this membrane through action of Na,K-ATPase. (*B*) Monensin (Mon) transports Na^+^ down the Na^+^ gradient. (*C*) Pb^2+^ is transported out. The net result of processes occurring in (*B*) and (*C*) is the electroneutral exchange of an intracellular Pb^2+^ and an OH^–^ for an external Na^+^.

Additional factors to consider are illustrated in Figure 16 and relate to the fact that monensin is an effective ionophore for sodium ions (Na^+^) compared with potassium ions (K^+^) (Henderson et al. 1969; Kinnally et al. 1991; Pressman 1968). In addition, near a membrane surface, the acid dissociation constant (pK*_a_*) for monensin is 6.85, and the Na^+^ dissociation constant (pK_N_*_a_*) is 5.00 (Hamidinia et al. 2002), whereas the *in vivo* concentrations of hydrogen ions (H^+^) and Na^+^ in extracellular volumes are about 10^–7^ M and 10^–3^ M, respectively. The p*K* values were obtained in solutions of 80% methanol in water, an environment that mimics a membrane–water interface in terms of polarity (Pfeiffer et al. 1983; Taylor et al. 1985, 1993). Furthermore, at physiologic pH, it appears that the predominant species by which monensin transports Pb^2+^ is the mixed complex monensin·Pb·OH (Hamidinia et al. 2002). Thus, when acting to deliver intracellular Pb^2+^ to extracellular DMSA, it is probable that the compound enters cells as the species monensin·Na, leaves as the mixed complex, and so in effect catalyzes an exchange of extracellular Na^+^ for intracellular PbOH^+^ (equivalent to exchanging an extracellular Na^+^ and an H^+^ for an intra-cellular Pb^2+^) (Figure 16). Given that the external/internal Na^+^ concentration gradient is maintained by Na,K-ATPase, the presence of monensin partially couples the release of intra-cellular Pb^2+^ to ATP hydrolysis, giving a direction and a driving force to the process. This may also help explain why the presence of monensin enhances the effectiveness of DMSA at removing Pb. However, it should also be pointed out that monensin may act in a more indirect way to enhance the action of DMSA. One possibility relates to the depletion of Pb from bone, where monensin might increase the rate of bone turnover. Were that to occur, access of Pb to DMSA would possibly be increased without a requirement for direct Pb^2+^ transport mediated by the ionophore.

A final point arises upon comparing an aspect of the present data with those from a previous study in which monensin alone was administered to Pb-intoxicated rats (Hamidinia et al. 2002). The earlier study showed that monensin alone accelerated the removal of Pb from several tissues, compared with no treatment, whereas in the present study this was seen only in heart tissue (Figure 1). In the present study, Pb was released more efficiently under the no-treatment condition than was reported earlier (Hamidinia et al. 2002), even though the design of both studies was very similar. These differences related to how the rats were housed: during the earlier study (Hamidinia et al. 2002), they were housed individually and in metabolic cages (i.e., standing on metal gratings), which are both considered to be stressful circumstances; in the present study rats were housed in pairs, in plastic cages containing normal bedding. When viewed collectively, these considerations suggest that the mechanism(s) that remove Pb under no-treatment conditions are less efficient in stressed rats and that monensin used alone is more efficient when organ Pb levels are higher. The later point is of interest here because it implies that monensin alone may be even more effective at higher levels of Pb intoxication and that other Pb ionophores having a higher affinity for Pb may also be more effective. These possibilities can be tested experimentally and are under investigation.

## Figures and Tables

**Table 1:** Effects of Pb administration on the concentrations (nmol/g) of selected elements in rat tissues.

	Tissue					
Element	Kidney	Liver	Skeletal muscle	Heart	Brain	Femur
Pb	29.8*	5.77*	0.073*	0.163*	1.19*	203*
	0.20	0.20	ND	0.005	0.05	ND
Physiologic						
Ca	897*	574	1,318*	522*	2,418	4,025,000*
	12,775	669	1,193	903	1,327	3,220,000
Co	1.24	0.352	0.025	0.523	0.093	0.130
	NR	NR	NR	NR	NR	NR
Cu	62.1*	51.1	13.6	101*	38.7*	5.29*
	96.9	59.2	14.0	78.4	33.4	45.5
Fe	524*	1,534*	133*	1,049	239*	771
	1,112	1,003	191	1,010	315	702
Mg	6,627	8,203*	10,028	8,885*	6,561*	117,300
	7,365	6,665	9,998	8,023	6,048	91,750
Mn	13.1*	32.0*	1.02*	7.34*	7.12	6.44*
	19.8	37.3	2.40	10.32	8.83	52.8
Mo	2.21	4.10	0.083	0.492	0.299	0.187
	NR	NR	NR	NR	NR	NR
Zn	295	472*	162*	315*	247*	1,844
	327	329	284	246	180	1,743
Nonphysiologic						
As	0.035	0.444	0.121	0.437	0.071	0.207
	NR	NR	NR	NR	NR	NR
Cr	0.012	0.004	0.033	0.886	0.066	ND
	NR	NR	NR	NR	NR	NR
Cd	0.082	0.062	0.003	0.005	0.005	0.006
	NR	NR	NR	NR	NR	NR
Ni	0.070	0.029	0.097	0.420	0.080	ND
	NR	NR	NR	NR	NR	NR
Sr	0.088	0.061	0.313	0.110	0.277	56.3
	NR	NR	NR	NR	NR	NR

Abbreviations: ND, not detected in this study; NR, not reported. The upper value for each element is from the present study and was determined after the rats had been given 100 ppm Pb^2+^ in their drinking water for 3 weeks (see “Materials and Methods”). The lower value for each element is from the literature and was determined using rats that had not been given Pb: values for Pb are from Hamidinia et al. (2002), and values for the other elements are from Naveh et al. (1987).

* Values obtained during this study that are statistically different (*p* < 0.05) from the corresponding literature value.
